# Preoperative cerebrospinal fluid cytokine levels and the risk of postoperative delirium in elderly hip fracture patients

**DOI:** 10.1186/1742-2094-10-122

**Published:** 2013-10-07

**Authors:** Dunja Westhoff, Joost Witlox, Leo Koenderman, Kees J Kalisvaart, Jos F M de Jonghe, Mireille F M van Stijn, Alexander P J Houdijk, Inge C M Hoogland, Alasdair M J MacLullich, David J van Westerloo, Diederik van de Beek, Piet Eikelenboom, Willem A van Gool

**Affiliations:** 1 Department of Neurology, Academic Medical Center/University of Amsterdam, PO box 22660, 1100 DD Amsterdam, the Netherlands; 2 Department of Geriatrics, Medical Center Alkmaar, Alkmaar, the Netherlands; 3 Department of Surgery, Medical Center Alkmaar, Alkmaar, the Netherlands; 4 Department of Respiratory Medicine, University Medical Center Utrecht, Utrecht, the Netherlands; 5 Department of Geriatric Medicine, Kennemer Gasthuis, Haarlem, the Netherlands; 6 Department of Intensive Care Medicine, University Medical Center Leiden, Leiden, the Netherlands; 7 GGZinGeest, Amsterdam, the Netherlands; 8 Edinburgh Delirium Research Group, Geriatric Medicine Unit, University of Edinburgh, Edinburgh, Scotland

**Keywords:** Delirium, Cytokines, Neuroinflammation, Cerebrospinal fluid, Neurodegeneration, Hip fracture

## Abstract

**Background:**

Aging and neurodegenerative disease predispose to delirium and are both associated with increased activity of the innate immune system resulting in an imbalance between pro- and anti-inflammatory mediators in the brain. We examined whether hip fracture patients who develop postoperative delirium have altered levels of inflammatory mediators in cerebrospinal fluid (CSF) prior to surgery.

**Methods:**

Patients were 75 years and older and admitted for surgical repair of an acute hip fracture. CSF samples were collected preoperatively. In an exploratory study, we measured 42 cytokines and chemokines by multiplex analysis. We compared CSF levels between patients with and without postoperative delirium and examined the association between CSF cytokine levels and delirium severity. Delirium was diagnosed with the Confusion Assessment Method; severity of delirium was measured with the Delirium Rating Scale Revised-98. Mann–Whitney U tests or Student t-tests were used for between-group comparisons and the Spearman correlation coefficient was used for correlation analyses.

**Results:**

Sixty-one patients were included, of whom 23 patients (37.7%) developed postsurgical delirium. Concentrations of Fms-like tyrosine kinase-3 (*P*=0.021), Interleukin-1 receptor antagonist (*P*=0.032) and Interleukin-6 (*P*=0.005) were significantly lower in patients who developed delirium postoperatively.

**Conclusions:**

Our findings fit the hypothesis that delirium after surgery results from a dysfunctional neuroinflammatory response: stressing the role of reduced levels of anti-inflammatory mediators in this process.

**Trial registration:**

The Effect of Taurine on Morbidity and Mortality in the Elderly Hip Fracture Patient.

Registration number: NCT00497978. Local ethical protocol number: NL16222.094.07.

## Background

Delirium, an acute and fluctuating impairment in attention and cognition, is the most common complication in hospitalized older people [1]. Delirium is associated with an increased risk of death, institutionalization, and dementia, independent of important confounders [2]. The pathophysiological mechanism of delirium is largely unknown, and a multifactorial etiology has been suggested [3]. Multiple lines of evidence indicate that a maladaptive neuroinflammatory response in the brain likely plays an important role in the development of delirium [4,5]. Several precipitating factors for delirium have been described, such as infection, malnutrition, fractures, and surgery [6]. All of these factors share an increased systemic production of cytokines [7,8]. Aging and cognitive impairment are the major predisposing factors for the development of delirium, and both have been associated with altered activity of the innate immune system of the central nervous system (CNS), resulting in an imbalance between pro- and anti-inflammatory cytokines [9]. Through the different pathways that serve immune-to-brain communication, a systemic inflammatory response may well lead to activation of microglia, the innate immune cells of the CNS. Activated microglia secrete several immune factors, such as cytokines and chemokines. In the elderly, microglia show a more reactive phenotype and release increased quantities of cytokines in the brain after peripheral stimulation [9]. This microglial response may be less well-regulated due to reduced cholinergic feedback in older persons, mediating the detrimental effects on prognosis [10].

Elderly patients with hip fracture are especially vulnerable, since the fracture itself and subsequent surgery are two events leading to a systemic response [11]. So far, no association has been shown between preoperatively measured pro-inflammatory cytokines in serum and the incidence of delirium after hip surgery in hospitalized elderly [12-14]. In contrast, one study did find a significant association between lower levels of the circulating anti-inflammatory cytokine Interleukin-1 receptor antagonist (IL-1ra) and delirium [13]. This might suggest that delirium is perhaps not so much the result of an increased pro-inflammatory state, but rather a consequence of reduced anti-inflammatory activity.

In this exploratory study, we hypothesized that elderly hip fracture patients with altered CNS cytokine profiles before surgery, indicating either increased pro-inflammatory or reduced anti-inflammatory activity, are specifically at risk for developing delirium postoperatively. We studied this hypothesis by analyzing a range of pro- and anti-inflammatory markers in cerebrospinal fluid (CSF) in elderly patients undergoing emergency hip surgery.

## Methods

### Ethical consideration

Was conducted in accordance with the guidelines of Good Clinical Practice. All patients gave written informed consent.

### Patients and clinical outcomes

Patients were participants in a double blind randomized study comparing effectiveness of taurine *versus* placebo in reducing morbidity and 1-year mortality in elderly hip fracture patients. During 1 year, all patients of 75 years or older who were admitted for surgical repair of a hip fracture in a teaching hospital in Alkmaar, the Netherlands, were checked for eligibility. Patients were excluded if they had no acute trauma, received total hip prosthesis, had a pathological fracture, were not willing or not capable (for example, dementia, aphasia, coma) to provide consent, or had contraindications regarding the administration of taurine (that is, renal failure defined as a creatinine clearing <30 mL/min). Written informed consent was obtained after eligibility was checked and the patient had been informed. Since all participants were at high risk of delirium (75 years or older, acute hospital admission), patients received routine care with prophylactic treatment of 0.5 mg haloperidol, three times daily from admission until postoperative day 3, unless contraindications were present [15]. Baseline assessment was completed within 12 h after submission, before surgery, including assessments of cognitive functioning, visual impairment, severity of acute illness, depression, activities of daily living, and risk factors, presence and severity of delirium as reported previously in more detail [2,16]. Medical records were inspected and patients and proxies were interviewed on prefracture functioning and demographic factors, including home situation and low or high educational level. Blood was drawn preoperatively to assess C-reactive protein (CRP), erythrocyte sedimentation rate (ESR) and IL-6 as a measure of systemic inflammation.

The main outcome was postoperative delirium, defined according to the Confusion Assessment Method (CAM) [17]. Presence and severity of delirium were assessed daily until the fifth postoperative day. Delirium severity was measured using the Delirium Rating Scale Revised-98 (DRS-R-98) [18]. In case of a positive CAM score, assessments were continued at least until the CAM was negative for 3 consecutive days or discharge. Severity of postoperative delirium was defined as the highest DRS-R-98 score.

### Cerebrospinal fluid samples and chemo- and cytokines

CSF samples were collected during canulation for the introduction of spinal anesthesia, prior to administration of any anesthetic. Lumbar punctures were performed with a 25-gauge needle between the L3-L4 or L4-L5 intervertebral space. A withdrawal protocol was used to standardize the handling of the CSF samples. In each patient 13 mL of CSF was collected in polypropylene tubes which were transported to the laboratory within 15 min after withdrawal. No later than 15 min after arrival at the laboratory the CSF samples were centrifuged at 1,800 g for 10 min at 4°C and aliquoted into polypropylene tubes that were stored at -20°C. The next day samples were transferred to -80°C.

To assess cytokine and chemokine levels, we used Luminex® technology: the Human Cytokine and Chemokine Panel, a premixed multiplex analysis (Milliplex, Millipore, Billerica, MA, USA). Based on previous experience with this assay, CSF samples were diluted 10 times. We determined 42 cytokines and chemokines: epidermal growth factor (EGF), eotaxin, fibroblast growth factor 2 (FGF-2), FMS-like tyrosine kinase 3 ligand (Flt-3L), Fractalkine, granulocyte colony stimulating factor (G-CSF), granulocyte macrophage colony stimulating factor (GM-CSF), growth-regulated oncogene (GRO), Interferon (IFN) α2, IFN-γ, IL-1ra, IL-1α, IL-1β, IL-2, soluble IL-2 Receptor alpha (sIL-2Ra), IL-3, IL-4, IL-5, IL-6, IL-7, IL-8, IL-9, IL-10, IL-12p40, IL-12p70, IL-13, IL-15, IL-17, Interferon gamma-induced protein 10 (IP-10), monocyte chemotactic proteins-1 and 3 (MCP-1, MCP-3), macrophage-derived chemokine (MDC), macrophage inflammatory proteins 1α and 1β (MIP-1α, MIP-1β), platelet-derived growth factors AA and AB/BB (PDGF-AA, PDGF-AB/BB), regulated and normal T-cell expressed and secreted (RANTES), soluble CD40 ligand (sCD40L), transforming growth factor α (TGF-α), tumor necrosis factors α and β (TNF-α and β), and vascular endothelial growth factor (VEGF).

### Analysis

We first compared preoperative CSF cytokine levels and baseline risk factors between patients with or without postoperative delirium. Next we also investigated the association between preoperative CSF cytokine levels and delirium severity. Statistics were performed using SPSS (SPSS for Windows, version 20, IBM Corporation, Armonk, NY, USA) or GraphPad Prism (GraphPad Software, version 5.01, La Jolla, CA, USA). Quantitative variables are presented as mean with standard deviation (SD) or median with interquartile range (IQR). Categorical variables were analyzed using Chi-Square or Fisher Exact tests. Continuous variables were tested with Mann–Whitney U tests or Student t-tests depending on sample size and distribution of the data. The assumption of normality was tested with the Kolmogrov-Smirnov test. Since cytokine data were significantly skewed, Mann–Whitney U tests were employed. Spearman’s correlation was used for correlation analysis. Statistical significance was set to *P* ≤0.05. Because of the exploratory nature of this study, correction for multiple comparisons was not applied.

## Results

**Figure 1:**
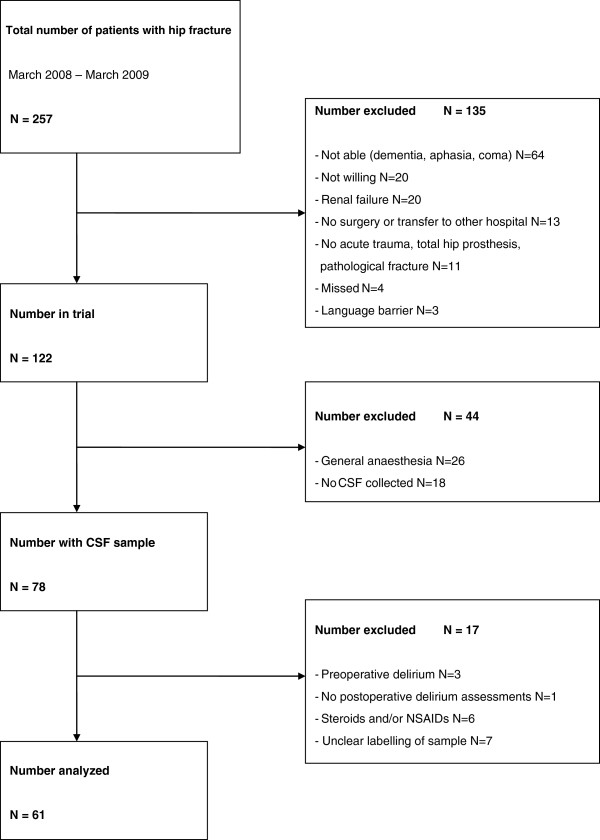
Flowchart of study inclusion.

From March 2008 to March 2009, 122 of 257 hip fracture patients fulfilled criteria for participation and provided consent (Figure 1). Of these 122 patients, 26 patients received general anesthesia and in 18 patients CSF collection could not be performed due to logistical limitations. Subsequently, 17 additional patients were excluded: samples were not labeled clearly (*n*=7), use of steroids and/or non-steroidal anti-inflammatory drugs (NSAIDs) (*n*=6), preoperative delirium (*n*=3), no postoperative delirium assessment (*n*=1), leaving 61 patients for analysis in this exploratory study. Twenty-three of these 61 patients (37.7%) developed postsurgical delirium. Median time of delirium onset after operation was 1 day (IQR 1.0-2.0), and 20 patients (87%) developed delirium within 2 days of surgery.

The characteristics of patients with and without postoperative delirium are shown in Table 1. Patients who developed delirium showed more signs of cognitive impairment, both immediately preoperative (MMSE) and before hospital admission (IQCODE-N). Additionally, these patients were more dependent on caregivers with regard to the activities of daily living (ADL) and instrumental ADL (IADL) functioning. Furthermore, patients who developed postoperative delirium were more severely ill prior to surgery as measured with the American Society for Anesthesiology classification system. With the exception of serum urea and creatinine levels [19], other baseline variables including serum parameters of liver function, renal clearance, creatine kinase, hemoglobin concentration, and white blood cell count between patients who did or did not develop postoperative delirium were similar (data not shown). Serum levels of CRP and ESR between patients with and without postoperative delirium were similar (*P*=0.354 and *P*=0.589, Table 2). Preoperative serum IL-6 was significantly higher in patients with postsurgical delirium (*P*=0.021).

**Table 1:** Baseline characteristics of patients with and without postoperative delirium

	**No delirium**	**Delirium**	***P *value**
	***n*=38 (62%)**	***n*=23 (38%)**	
Age (years)	82.9 ±4.5	84.6 ±5.2	0.18
Gender n/N (% female)	26/38 (68.4)	16/23 (69.9)	0.93
Living independently, n/N (%)	25/38 (65.8)	15/23 (65.2)	0.68
Low educational level, n/N (%)	13/36 (36.1)	7/22 (31.8)	0.78
Visual impairment^a^, n/N (%)	2/38 (5.3)	1/21 (4.8)	0.93
APACHE II^b^ score	13 (11–13)	13 (12–14)	0.20
ASA^c^ group, n/N (%)			0.04
Group I;	17/38 (44.7)	4/23 (17.4)	
II;	17/38 (44.7)	12/23 (52.2)	
III;	4/38 (10.5)	7/23 (30.4)	
Number of co-morbid diseases	1.0 (1.0-2.0)	2.0 (1.0-2.0)	0.26
Number of medications at home	2.5 (1.0-5.0)	4.0 (3.0-5.0)	0.06
IQCODE-N^d^ score	3.2 (3.0-3.4)	3.8 (3.3-4.1)	<0.001
IQCODE-N^d^ score >3.6, n/N (%)	4/37 (10.8)	13/21 (61.9)	<0.001
MMSE^e^ score	25.6 ±2.5	23.2 ±3.6	0.005
MMSE^e^ score <24, n/N (%)	8/37 (21.6)	11/22 (50.0)	0.04
GDS^f^ score	2.0 (1.0-3.0)	2.0 (1.0-3.0)	0.96
BI^g^ score	19.0 (17.9-20.0)	18.0 (14.8-20.0)	0.03
Lawton IADL^h^ score	10.0 (8.0-14.7)	14.5 (11.3-17.8)	0.03

Values are expressed as means ± SD or median (IQR), n/N is number with characteristic/total number, (%) is percentage.

^a^Visual impairment measured with the standardized Snellen test for visual impairment and defined as binocular near vision worse than 20/70 after correction.

^b^APACHE II: Acute Physiological and Chronic Health Evaluation II, range 0 (no acute health problems) to 70 (severe acute health problems).

^c^ASA: American Society of Anesthesiologists physical status classification system, range 1 (normal health) to 5 (moribund).

^d^IQCODE-N: Informant Questionnaire on Cognitive Decline in the Elderly - Short Form, range 16 (cognitive improvement) to 80 (severe cognitive decline), mean score >3.6 indicates cognitive decline.

^e^MMSE: Mini Mental State Examination, range 0 (severe cognitive impairment) to 30 (no cognitive impairment), score <24 indicates cognitive impairment.

^f^GDS: Geriatric Depression Scale, range 0 (depression not likely) to 15 (depression very likely).

^g^BI: Barthel Index of Activities of Daily Living, range 0 (severe disability) to 20 (no disability).

^h^Lawton IADL: Lawton Instrumental Activities of Daily Living scale, range 8 (no disability) to 31 (severe disability).

**Table 2:** Preoperative serum biomarker levels of patients with and without postoperative delirium

**Biomarker**	**No delirium**	**Delirium**	***P *value**
	***Median (IQR)***	***Median (IQR)***	
IL-6	23.16 (9.51-44.10)	48.13 (24.85-55.74)	0.021
	*N=37*	*N=23*	
CRP^*^	3.00 (1.00-6.50)	4.00 (1.00-8.00)	0.354
	*N=17*	*N=14*	
ESR^#^	14.00 (8.00-23.50)	15.00 (7.00-23.00)	0.589
	*N=37*	*N=23*	

^*^CRP, C-reactive protein; ^#^ESR, erythrocyte sedimentation rate.

In CSF, eotaxin, IL-12p40, MIP1α, MIP1β, and VEGF were below limit of detection in all samples. EGF, FGF2, Fractalkine, GM-CSF, GRO, IL-1β, IL-2, IL-3, IL-4, IL-5, IL-7, IL-9, IL-10, IL-12p70, IL-13, IL-17, MDC, PDGF-AB/BB, sIL-2ra, TNF-α, and TNF-β were detectable in CSF of <50% of patients only. Patients who developed postoperative delirium were less likely to have detectable levels of IL-3 (44.7% *vs.* 17.4%, *P*=0.05). Except for IL-3, no differences were found in detectability of cytokines per group (Table 3). Because of these limited numbers and the resulting reduced power, no further analyses were performed on these variables.

**Table 3:** Number of patients with detectable preoperative CSF cytokine concentrations

**Cytokine/chemokine**	**No delirium (N=38)**	**Delirium (N=23)**	***P *value**
	***n (%)***	***n (%)***	
EGF	4 (10.5)	7 (30.4)	0.083
FGF-2	4 (10.5)	3 (13.0)	1
Fractalkine	8 (21.0)	7 (30.4)	0.541
GM-CSF	2 (5.3)	1 (4.3)	1
GRO	2 (5.3)	0 -	0.522
IL-1β	1 (2.6)	1 (4.3)	1
IL-2	3 (7.9)	4 (17.4)	0.409
IL-3	17 (44.7)	4 (17.4)	0.050
IL-4	11 (28.9)	3 (13.0)	0.214
IL-5	15 (39.5)	6 (26.1)	0.406
IL-7	14 (36.8)	5 (21.7)	0.264
IL-9	1 (2.6)	0 -	1
IL-10	7 (18.4)	2 (8.7)	0.462
IL-12p70	1 (2.6)	1 (4.3)	1
IL-13	15 (39.5)	7 (30.4)	0.586
IL-17	1 (2.6)	0 -	1
MDC	8 (21.0)	3 (13.0)	0.510
PDGF-AB/BB	1 (2.6)	0 -	1
sIL-2Ra	7 (18.4)	4 (17.4)	1
TNF-α	12 (31.6)	7 (30.4)	1
TNF-β	4 (10.5)	3 (13.0)	1
MDC	8 (21.0)	3 (13.0)	0.510

Preoperative CSF concentrations of cytokines that were measurable in at least 50% of all samples are presented in Table 4. Concentrations of Flt-3L (*P*=0.021), IL-1ra (*P*=0.032), and IL-6 (*P*=0.005) were significantly lower in patients who developed delirium postoperatively. Concentrations of IL-15 were lower (*P*=0.060) and IP-10 were higher (*P*=0.064) in the patients who developed delirium compared to those who did not develop delirium, though these differences did not reach statistical significance. Restricting our comparisons with controls to only those patients who developed delirium shortly after surgery (<48 h) generally did not affect any of the findings mentioned above. Concentrations of CSF cytokines were not related to treatment allocation in taurine of placebo groups.

**Table 4:** Preoperative CSF cytokine levels of patients with and without postoperative delirium

**Cytokine/chemokine**	**Concentration (pg/mL)**		***P *value**
	**No delirium (N=38)**	**Delirium (N=23)**	
	***Median (IQR)***	***Median (IQR)***	
Flt-3L	20.16 (18.2-29.5)	16,54 (12.2-20.2)	0.021
G-CSF	31.26 (14.7-55.8)	23.15 (14.7-33.9)	0.233
IFN-α2	31.70 (21.1-41.1)	24.41 (17.7-34.1)	0.136
IFN-γ	1.61 (1.6-2.4)	1.61 (1.6-2.4)	0.336
IL-1α	0.28 (0.2-2.3)	0.78 (0.1-1.3)	0.170
IL-1ra	6.90 (2.9-6.9)	2.90 (2.9-6.9)	0.032
IL-6	0.99 (0.2-2.2)	0.21 (0.1-0.7)	0.005
IL-8	28.40 (22.7-42.5)	25.25 (19.4-37.6)	0.308
IL-15	3.47 (1.4-4.5)	2.79 (1.4-3.5)	0.060
IP-10	270.84 (183.1-350.6)	334.21 (241.0-590.0)	0.064
MCP-1	312.67 (257.2-422.6)	301.79 (241.6-389.5)	0.634
MCP-3	19.4 (12.2-25.2)	19.4 (19.4-25.2)	0.375
PDGF-AA	8.04 (5.6-10.3)	6.67 (5.4-9.0)	0.267
RANTES	3.50 (0.1-3.5)	0.08 (0.1-3.5)	0.290
sCD40-L	1.14 (0.06-1.1)	0.06 (0.06-1.1)	0.263
TGF-α	1.99 (1.4-3.2)	1.99 (1.4-2.6)	0.411

We explored the relationship between age, cognitive function, and CSF cytokine variables. No significant correlations were found between age, baseline cognitive function (MMSE), pre-existent cognitive impairment (IQCODE-N), and levels of CSF cytokines. Stratification of the MMSE and IQCODE-N according to the abovementioned cutoff scores did not reveal different outcomes (data not shown). As levels of cytokines tend to fluctuate over time we investigated if time from admission to surgery influenced CSF cytokine levels. Mean time from admission to surgery was 19 h (SD ± 10.0). The time between admission and surgery was not associated with levels of CSF cytokines or their ratios. In contrast, both the incidence (*P*=0.05) and the severity (Spearman’s rho= 0.32, *P*=0.009) of postoperative delirium were influenced by the time patients waited for hip surgery.

## Discussion

We explored the association between the occurrence of postoperative delirium and preoperative cytokine and chemokine levels in the CSF. Despite the limited power of this exploratory study, we found significantly lower levels of Flt-3L, IL-1ra, and IL-6 and distinct trends for lower levels of IL-15 and higher levels of IP-10 in CSF of patients with postoperative delirium. Although the nature of the present study is clearly explorative, these findings are consistent with the hypothesis that delirium after surgery results of neuroinflammatory changes [4,10,20].

IL-1ra is an inhibitor of the pro-inflammatory cytokines IL-1β and IL-1α and is naturally present in the brain [21]. Reduced CSF levels of this inhibitor are therefore associated with an increased inflammatory state of the CNS. This is illustrated by the fact that intrathecal levels of IL-1ra are significantly lower in patients with Alzheimer’s disease (AD) [22] and in patients with peripheral inflammatory diseases such as rheumatic arthritis [23], similar to the patients who developed delirium in the current study.

Flt-3L is an early acting cytokine that potently stimulates the proliferation, differentiation and mobilization of hematopoietic precursor cells into macrophages, osteoclasts, microglial, and dendritic cells [24,25]. The dendritic cell represents a cellular component of an immune circuit between the brain immune system and the periphery [26]. The introduction of adenovirus expressing Flt3L in animals enhanced the formation of dendritic cells [27], while inhibition of the Flt3 receptor in an experimental mouse model of MS led to apoptosis of dendritic cells and aggravation of symptoms [28]. In the healthy brain, the Flt-3 receptor is highly expressed in neurons of the hippocampus and cerebellum [29], while Flt-3Ligand is abundantly expressed in the pyramidal cells of the hippocampus [29], an area important for memory and spatial navigation. In CSF of AD patients, low levels of Flt-3L have been observed [30]. Lower levels of Flt-3L thus may play a role in the increased susceptibility to infection [31] and chronic inflammatory states [32] in older persons [33], and in cognitive decline.

The trend towards preoperatively increased IP-10 concentration in patients who developed delirium postoperatively is interesting since IP-10 plays a pivotal role in the immune system as an important regulator of the growth of immature hematopoietic progenitor cells, and in T-cell migration. IP-10 is secreted by astrocytes and microglia, and elevations of this chemokine in CSF of patients with mild cognitive impairment and mild AD have been described [34]. Immunohistochemically, IP-10 expression is upregulated in AD brain sections compared to controls [35]. In the brain tissue of patients with HIV-associated dementia, a significant increase in neuronal cell death was observed when *in vitro* brain tissue was exposed to IP-10 [36]. The trend towards higher IP-10 levels in patients with postoperative delirium suggests a role of this chemokine in an active chronic-inflammatory process. Taken together, lower levels of IL-1ra, Flt-3L, and IP10 might reflect a skewing towards a pro-inflammatory phenotype in the brain contributing to the development of delirium postoperatively.

In this descriptive study it is surprising to find that lower levels of IL-6 as well as IL-15 were associated with the development of postoperative delirium.

IL-6 is a critical cytokine connecting innate and acquired immunity, and is vital for induction of both peripheral and central defence against injury and inflammation [37]. It is secreted by immune cells, including microglia, in response to danger signals. Although IL-6 is often related to inflammation, it also contributes in the normal function of the brain [38]. Studies with IL-6 knockout mice show an important role for IL-6 in sleep-wake behavior [39], emotional reactivity [40], sickness behavior [41], and learning and memory [42]. Furthermore, it is important for adult neurogenesis [43] and gliogenesis [44]. IL-6 levels are detectable in the serum of older subjects, even in the absence of illness or inflammation [45]. This increase might contribute to the development of frailty and the predisposition to inflammatory diseases [46]. IL-6 is increased also in amyloid plaques or brain tissue extract of AD patients [47], suggesting that IL-6 is associated with an increased neuroinflammatory state in AD [48]. Amyloid-β (Aβ) induces massive gliosis and triggers IL-6 production. However, *in vivo* studies with AD transgenic mouse models suggest a protective role of IL-6, since it can induce differentiation of microglia into phagocytic macrophages capable of degrading Aβ [49,50]. Although IL-6 seems to be overexpressed in brains of AD patients, limited observational data suggest that IL-6 levels are not increased in the CSF of AD patients [34,51]. In patients with delirium, like Alzheimer’s patients, blood and brain IL-6 are higher compared to controls [52-54]. We found that patients with postoperative delirium have higher levels of serum IL-6, which is conform the systemic inflammatory hypothesis of delirium. Higher IL-6 levels in preoperative serum in association with postoperative delirium, in the absence of such a relation in CSF, is difficult to explain. One can speculate that increased serum IL-6 levels may be an early manifestation of an increased inflammatory reaction in patients prone to develop delirium 24 h later on average. A previous study, in which rats were subject to femur fracture, showed elevated systemic cytokines 24 h after injury while brain cytokines did not differ from controls [55]. Increased systemic, serum cytokine levels may precede an increased neuroinflammatory response associated with delirium.

The trend of lower CSF levels of IL-15 is difficult to interpret. IL-15 is a cytokine that is produced by astrocytes and microglia in response to danger signals such as toxins and pro-inflammatory cytokines [56]. It actively modulates microglial reactivity, controlling additional cytokine, and chemokine release [57], but no consensus has been reached about the clinical consequences of this modulation [58]. Follow-up studies should focus on investigating the role of these two cytokines as the current state of knowledge does not enable us to explain the differences observed with regard to IL-6 and IL-15.

Previous studies have shown that in elderly hip fracture patients, blood levels of inflammatory cytokines are associated with delirium [12-14]. However, these studies included both delirious and non-delirious patients at the time of sampling, or evaluated the postoperative time-course of cytokines in delirium and controls. Two studies did measure preoperative cytokines in non-delirious patients. In one study, in mainly healthy older people undergoing elective hip replacement [12], the absence of differences in serum cytokines between patients with and without postoperative delirium, can be taken to reflect the absence of acute illness. The second study only included acutely ill older persons, and found that low serum levels of neuroprotective factors IGF-I and IL-1ra were associated with delirium [13]. One earlier preliminary study examined CSF cytokines in hip fracture patents, and showed higher preoperative IL-8 levels in hip fracture patients with delirium as compared to non-delirious controls [59]. However, this study included patients with pre- and postoperative delirium at the time of CSF collection, and thus could not determine whether higher CSF IL-8 should be regarded as a 'risk factor’ for delirium or as a 'marker’ of delirium. Our findings are based on a larger sample of patients all without delirium at the time of CSF sampling.

The present data suggest that delirium is perhaps not the result of an increased pro-inflammatory state of the CNS, but rather is caused by an impaired anti-inflammatory response. Without these regulatory cytokines, the brain may be subject to unrestrained neuro-inflammation, which may clinically manifest itself as delirium. However, the results of this exploratory study are not sufficiently strong enough to use the observed cytokine profiles for predicting delirium in postsurgical elderly. Inflammation is a highly complex and dynamic process during which many different cytokines and numerous other effectors interact. The state of inflammation is thus not likely to be dependent on the net effect of individual cytokines only, but on the balance between pro- and anti-inflammatory mediators [9]. Moreover, inflammatory markers CRP and ESR did not significantly differ between groups, which is consistent with previous results [12,14,54]. This finding shows that the systemic inflammatory environment does not necessarily reflect inflammation in the CNS and *vice versa*.

Although we employed a sensitive method to quantify cytokines [60], levels of 5 cytokines were below the detection limit, and 21 cytokines were detectable in a minority of patients only. This could be due to low levels of these cytokines in the CSF or perhaps be related to compartmentalization of cytokines in the brain extracellular space [61], and/or to their short half-life [62]. Mean time between admission and sampling was 19 h. One could argue that cytokine levels that changed following fracture, could have normalized in this time-window. However, several studies show that CSF cytokine levels remain altered at least for 20 h, possibly even extending to 96 h after the insult [63,64]. There are several issues that deserve further comment. We deliberately choose not to include adjustments for baseline differences in our exploratory analyses because in doing so the mediating effect of changes in cytokine profiles might be obscured. The question of whether changes in the blood–brain barrier or specific effects of medication may have played a role should be addressed in future studies, even though patients who used steroids and/or NSAIDs prior to hospital admission were excluded in our analyses. Delirium assessments took place several hours before CSF samples were collected. Despite close attention of the nursing staff, during this window, in theory, some patients could have developed preoperative delirium, and as consequence may have been falsely classified in the preoperative period. Time between hip fracture and subsequent surgery potentially represents an important confounder of the association between cytokines and postoperative delirium. We were not able to examine this since we do not have data on the time period between fracture and hospital admission. However, we did examine the influence of a related time period, namely the 'time between hospital admission and surgery’. All participants received routine care with low dose prophylactic haloperidol. Haloperidol has been reported to reduce both the severity of postoperative delirium [15] and levels of pro-inflammatory cytokines [65]. However, several studies that assessed the influence of haloperidol on cytokines in schizophrenic patients, did not find any effect on serum levels of IL-1ra, sIL2R, IL-6, and TNF-α after 6 weeks of treatment [66]. IL-2 levels did decrease after 8 weeks of treatment, although the mean dose in this study was 14.9 mg/day [66,67]. In CSF of schizophrenic patients, haloperidol treatment did not significantly alter levels of IL-6 and IL-2 [68]. One study examined levels of serum sIL-2r after a haloperidol challenge of 4 or 10 mg in normal, healthy controls, which showed no influence on serum levels of sIL-2r [69]. Patients in our study received much lower doses of haloperidol, 1.5 mg per day. Acute neuroleptic administration does not seem to influence cytokine levels at these low doses.

A role for neuroinflammation in the pathophysiology of delirium may provide new opportunities for clinical intervention. The poor long-term prognosis of delirium in elderly patients [2] and the evidence for prevention over management of delirium underlines the importance of adequate preventive measures [70]. Interventions that may ameliorate neuroinflammation could raise the reserve capacity of the brain and thereby the threshold for episodes of delirium. Controlled studies are necessary to validate a role of an impaired anti-inflammatory response in the pathophysiology of delirium and to investigate if delirium and its negative sequelae can be averted by targeting the neuroinflammatory balance.

## Conclusion

Our findings fit the hypothesis that delirium after surgery results from a dysfunctional neuroinflammatory response: this may partly be a consequence of reduced levels of anti-inflammatory mediators rather than exclusively an excess of pro-inflammatory cytokines. Because the brain is never exposed to only a single cytokine, future studies should focus on the balance between multiple pro- and anti-inflammatory mediators. The influence of the innate immune system in delirium pathophysiology offers scope for prevention by targeting neuroinflammation.

## Abbreviations

AD: Alzheimer’s disease; CAM: Confusion assessment method; CNS: Central nervous system; CRP: C-reactive protein; CSF: Cerebrospinal fluid; DRS-R-98: Delirium Rating Scale Revised-98; EGF: Epidermal growth factor; ESR: Erythrocyte sedimentation rate; FGF: Fibroblast growth factor; Flt-3L: FMS-like tyrosine kinase 3 ligand; G-CSF: Granulocyte colony stimulating factor; GM-CSF: Granulocyte macrophage colony stimulating factor; GRO: Growth-regulated oncogene; IFN: Interferon; IL-1ra: Interleukin-1 receptor antagonist; IP-10: Interferon gamma-induced Protein 10; IQCODE-N: Informant Questionnaire on Cognitive Decline in the Elderly; MCP: Monocyte chemotactic protein; MDC: Macrophage-derived chemokine; MIP: Macrophage inflammatory protein; MMSE: Mini mental state evaluation; NSAIDs: Non-steroidal anti-inflammatory drugs; PDGF: Platelet-derived growth factors; RANTES: Regulated and normal T-cell expressed and secreted; sCD40L: Soluble CD40 ligand; TGF: Transforming growth factor; TNF: Tumor necrosis factor; VEGF: Vascular endothelial growth factor.

## Competing interests

The authors declare that they do not have any competing interests.

## Authors’ contributions

JW, LK, KJK, PE, and WAvG designed the study. DW carried out multiplex analysis. DW and JW acquired and interpreted data, performed statistical analyses, and drafted the manuscript. MFMS and APJH acquired data. DvdB, AMJM, DJvW, and WAvG interpreted data. LK, KJK, PE, JFMJ, ICH, DvdB, AMJM, DJvW, and WAvG critically revised the manuscript. All authors read and approved the final version of the manuscript.
